# Low dose environmental radon exposure and breast tumor gene expression

**DOI:** 10.1186/s12885-020-07184-7

**Published:** 2020-07-28

**Authors:** Cheng Peng, Natalie DuPre, Trang VoPham, Yujing J. Heng, Gabrielle M. Baker, Christopher A. Rubadue, Kimberly Glass, Abhijeet Sonawane, Oana Zeleznik, Peter Kraft, Susan E. Hankinson, A. Heather Eliassen, Jaime E. Hart, Francine Laden, Rulla M. Tamimi

**Affiliations:** 1 Channing Division of Network Medicine, Brigham and Women’s Hospital, Boston, MA USA; 2 Department of Epidemiology, University of Louisville School of Public Health and Information Science, Louisville, KY USA; 3 Department of Epidemiology, Harvard T. H. Chan School of Public Health, Boston, MA USA; 4 Department of Pathology, Beth Israel Deaconess Medical Center, Harvard Medical School, Boston, MA USA; 5 Cancer Research Institute, Beth Israel Deaconess Cancer Center, Boston, MA USA; 6 Department of Biostatistics, Harvard T. H. Chan School of Public Health, Boston, MA USA; 7 Department of Biostatistics and Epidemiology, School of Public Health and Health Sciences, University of Massachusetts Amherst, Amherst, MA USA; 8 Department of Environmental Health, Harvard T. H. Chan School of Public Health, Boston, MA USA

**Keywords:** Low-dose radon, Tumor gene expression, Breast cancer risk

## Abstract

**Background:**

The International Agency for Research on Cancer classified radon and its decay-products as Group-1-human-carcinogens, and with the current knowledge they are linked specifically to lung cancer. Biokinetic models predict that radon could deliver a carcinogenic dose to breast tissue. Our previous work suggested that low-dose radon was associated with estrogen-receptor (ER)-negative breast cancer risk. However, there is limited research to examine the role of radon in breast cancer biology at the tissue level. We aim to understand molecular pathways linking radon exposure with breast cancer biology using transcriptome-wide-gene-expression from breast tumor and normal-adjacent tissues.

**Methods:**

Our study included 943 women diagnosed with breast cancer from the Nurses’ Health Study (NHS) and NHSII. We estimated cumulative radon concentration for each participant up-to the year of breast cancer diagnosis by linking residential addresses with a radon exposure model. Transcriptome-wide-gene-expression was measured with the Affymetrix-Glue-Human-Transcriptome-Array-3.0 and Human-Transcriptome-Array-2.0. We performed covariate-adjusted linear-regression for individual genes and further employed pathway-analysis. All analyses were conducted separately for tumor and normal-adjacent samples and by ER-status.

**Results:**

No individual gene was associated with cumulative radon exposure in ER-positive tumor, ER-negative tumor, or ER-negative normal-adjacent tissues at FDR < 5%. In ER-positive normal-adjacent samples, *PLCH*2—reached transcriptome-wide-significance (FDR < 5%). Gene-set-enrichment-analyses identified 2-upregulated pathways (MAPK signaling and phosphocholine biosynthesis) enriched at FDR < 25% in ER-negative tumors and normal-adjacent tissues, and both pathways have been previously reported to play key roles in ionizing radiation induced tumorigenesis in experimental settings.

**Conclusion:**

Our findings provide insights into the molecular pathways of radon exposure that may influence breast cancer etiology.

## Background

Radon—a natural radioactive gas originating from the decay series of uranium found in the Earth’s crust—accounts for a significant proportion of the annual effective dose of natural radioactivity [1], and have been extensively studied in relation lung cancer [2]. Radon is fat-soluble, tends to bio-accumulate in tissues with higher fat content such as the breast, has a relatively short half-life (~ 3.8 days), and undergoes rapid decay processes to emit alpha particles that may interact with biological tissues and macro-molecules [3, 4]. Similar to other ionizing radiation, radon and its decay products are mutagenic [4], as they could lead to single and double-strand DNA breaks [5], pyrimidine dimer formations [6], intra- and inter-chromosomal aberrations [7, 8] sister chromatid exchange [9, 10] micronuclei formation [11] and eventually genomic instability [5]. Residential radon exposures were associated with increased lung cancer risk among never smokers who carried genetic polymorphisms in DNA-repair genes [12]. Chromosomal abnormality was also observed in lung cancer patients exposure to radon and air pollution [13]. In areas with high radon background, increased levels of indoor radon concentrations were associated with chromosomal translocation and aberrations [14, 15]. Although limited by the number of cases, a suggestive association was observed between residential radon and higher risk of breast cancer, in a radon-prone area [16]. In the Nurses’ Health Study II (NHSII), VoPham et al. conducted a prospective analysis of radon exposure and incidence of invasive breast cancer, and demonstrated that cumulative radon exposure, even at low doses, was associated with higher risk of estrogen receptor (ER)-negative breast cancer [17]. However, the molecular mechanisms underlying the effects of radon on breast cancer risk are not fully understood. A better understanding of the biological pathways associated with low dose radon in population-based studies may provide important human evidence supporting the role of environmental exposure in breast carcinogenesis.

The study of transcriptome-wide gene expression profiling in breast tumor and normal-adjacent tissue offers a unique opportunity to uncover biological mechanisms linking environmental exposures such as radon and breast cancer risk. The breast tissue-specific transcriptome provides deep coverage of measurable mRNA transcripts, and provides a quantitative and systematic readout of the pathophysiological status of breast tissue that reflects the complex interplay between genetics and both endogenous and exogenous environmental influences. In breast cancer cell lines, ionizing radiation leads to differential gene expression in pathways related to apoptosis [18, 19] inflammation [19] oxidative stress [19, 20] DNA damage and repair [12, 19] and cell cycle regulation [19]. As tissue specific transcriptomes capture detailed levels of molecular activities, transcriptomic analyses of breast tumor and normal-adjacent tissues may provide a comprehensive assessment of biological mechanisms underlying radon related carcinogenesis.

In two large-scale prospective cohort studies in the United States, we investigated the association between radon concentrations at the participants’ addresses—estimated as cumulative averages up to the year of breast cancer diagnosis—and transcriptome-wide gene expression in breast tumor and normal-adjacent tissues. We hypothesized that breast cancer cases living in areas of higher radon concentration would have differential gene expression patterns in tumor and normal-adjacent tissues compared with women living in areas of lower concentrations. While differences in gene expression signatures in tumor tissues may provide information about radon-associated tumor initiation and progression; alterations in gene expression patterns in normal-adjacent tissues may reflect a larger field effect in the breast before carcinogenesis.

## Methods

### Study population

Our study includes participants from the Nurses’ Health Study (NHS) and the NHSII—both of which are large-scale prospective longitudinal cohorts of registered female nurses in the U.S. The NHS was established in 1976, when 121,701 women aged 30–55 years, completed and returned an initial questionnaire. In 1989, the NHSII was initiated, which enrolled 116,429 women, ages 25–42 years, who completed and returned an initial questionnaire. Participants have been followed via questionnaires mailed biennially to update information on exposure variables and ascertain outcomes [21]. The cumulative follow-up rates for NHS and NHSII were both greater than 90% [22]. Cases of invasive breast cancer were identified by participants’ responses to the biennial questionnaires from the start of follow-up (1976 NHS; 1989 NHSII) through 2012. Following a reported diagnosis, study investigators requested permission to review the participant’s medical records to confirm the diagnosis. Over 99% of breast cancer diagnosis were confirmed upon medical record review. For deceased participants, we contact next-of-kin for record review permission or link to cancer registries. With permission, we linked 96% of breast cancer cases to relevant medical records. The study protocol was approved by the institutional review boards of the Brigham and Women’s Hospital and Harvard T. H. Chan School of Public Health, and those of participating registries as required.

### Radon estimation

We collected participants’ residential addresses from biennial questionnaires updated since initial enrollment in NHS (1976) and NHSII (1989). Residential addresses were geocoded and spatially linked to the Laurence Berkeley National Laboratory U.S. county-level indoor radon exposure model using a geographic information system (GIS) ArcMap 10.3.1 (Esri, Redlands, CA) [17]. The radon exposure model was estimated using a Bayesian mixed-effect regression to predict annual average county-level radon concentrations derived from the short-term Environmental Protection Agency (EPA)/State Residential Radon Survey (SRRS) and long-term National Residential Radon Survey (NRRS) [23, 24]. We calculated radon concentration as a time-varying cumulative average for each participant, where radon concentrations from years prior to diagnosis were averaged and updated biennially until a breast cancer diagnosis [17]. Radon concentration was expressed as pCi/L based on the scale established by the EPA (1pCi/L = 37 Bq/m^3^ international unit).

### Gene expression measurements

We began to collect archived formalin-fixed paraffin-embedded breast cancer blocks for participants with primary incident breast cancer since 1993. Formalin fixed paraffin-embedded (FFPE) tumor blocks were retrieved from the pathology departments of treating hospitals [25]. We were able to obtain tissue microarrays (TMAs) from 5561 NHS and NHSII participants (Dana Farber / Harvard Cancer Center Tissue Microarray Core Facility, Boston, MA) [25, 26]. Prioritizing women with existing genetic and circulating biomarker measurements, we were able to perform tissue gene expression microarrays among 954 breast cancer cases. Participant characteristics were similar among breast cancer cases with and without gene expression measurement [27]. We extracted RNA from multiple cores of 1 or 1.5 mm taken from tumor (*n* = 1–3 cores) and normal-adjacent (*n* = 3–5 cores) tissues using the Qiagen AllPrep RNA isolation kit for FFPE. Normal-adjacent tissues were obtained > 1 cm away from the tumor edge. A detailed protocol has been published previously [28–30] (microarray data accession number: GSE115577). In brief, we measured gene expression using Affymetrix Glue Grant Human Transcriptome Array 3.0 (hGlue 3.0) for samples processed in 2012–2014 and Human Transcriptome Array 2.0 (HTA 2.0) microarray chips (Affymetrix, Santa Clara, CA, USA) for samples processed in 2015–2018. All microarrays were scanned with the GeneChip® Scanner 3000 7G (Affymetrix, Santa Clara, CA, USA). We normalized gene expression data using robust multi-array average (RMA; Affymetrix Power Tools (ATP)), and performed sample quality control using Affymetrix Power Tools probeset summarization based metrics [28, 29]. A total of 2160 FFPE samples were assayed. We excluded samples that failed quality control steps (*n* = 440), technical replicates (*n* = 139), samples with unknown IDs (n = 4). In total, 1577 samples (882 tumor tissues and 695 normal-adjacent tissues) from 954 invasive breast cancer cases passed quality control. For genes that were mapped by multiple probes, the most variable probe was selected to represent the gene. The hGlue 3.0 platform included 18,102 genes with unique Entrez ID and the HTA 2.0 platform included 25,023 genes with unique Entrez ID. In our current analysis, we included 17,791 genes that were in common among the two platforms (70% overlapped). We controlled for known technical variabilities (batch / plate) using *ComBat*—an empirical Bayes method used to control for known batch effects [31]. We further removed genes with low expression (< 25th percentile).

### Covariates and final dataset

We obtained information on potential risk factors for breast cancer via the biennial NHS and NHSII questionnaires (age, body mass index (BMI), hormone therapy (HT) use) or pathological reports (year of diagnosis). Median household income and region of residence were based on linking geocoded residential addresses with data from the U.S. Census Bureau [17]. ER status was determined via central review of breast tissue microarrays [25]. To calculate pack-year smoking, we multiplied smoking duration in years by packs of cigarettes smoked per day [32]. We restricted our analysis to women with complete exposure and covariate data, resulting in 943 breast cancer cases (874 tumor tissues, 687 normal-adjacent tissues; 1561 total samples; 618 tumor-normal-adjacent pairs).

### Statistical analysis

#### Single gene analysis

We evaluated the association of cumulative average radon concentrations and transcriptome-wide gene expression at each individual gene using covariate-adjusted linear regression via the R Bioconductor package linear models for microarray data (*limma*) [33]. County radon measurements ranged between 4 and 30. The surveys were designed using a population-based stratified random sampling scheme with the intent of being representative of the study area. Radon concentrations were dichotomized at 2 pCi/L (< 2 pCi/L: low; ≥2 pCi/L: high) based on the U.S. EPA Assessment of Risks from Radon in Homes that suggests remediation at 2 pCi/L based on the lifetime risk for lung cancer deaths [34, 35]. This cut point (2 pCi/L) is also in alignment with our previous findings reporting environmental radon exposure and breast cancer risk, where we observed statistically significant associations between radon in the highest quintile (74.9 Bq/m^3^ or 2.02 pCi/L) with ER-negative breast cancer risk [17]. As a sensitivity analysis, we modeled radon as a continuous exposure matrix. In each model, we adjusted for the following covariates selected a priori: age at diagnosis (continuous), year of diagnosis (continuous), menopausal status and HT use (post-menopausal not using / post-menopausal using / premenopausal or unknown), BMI (continuous), Census tract area-level socioeconomic status (continuous), study region (Northeast / Midwest / West / South) and surrogate variables generated from the transcriptome data (leek method) (Bioconductor *sva* package in R) [36]. Distributions of family, age at first birth and parity did not differ by radon exposure (≥ 2pCi/L vs. < 2pCi/L). We therefore did not include them in the regression models. We analyzed tumor and normal-adjacent tissues separately, and we stratified the analysis by ER status. A gene was considered to be significant transcriptome-wide if it reached the FDR-corrected *p*-value of *p*_*BH*_ *< 0.05* based on the method of Benjamini and Hochberg [37].

Among the top 10 most significant genes identified in tumor tissue, we performed sensitivity analyses and examined the associations between radon exposure and gene expression among never smokers and women smoked > 16 pack-years (16 pack-years is the population median among ever smokers). Given the latency period of radon exposure to cancer incidence, we also restricted radon exposure 5 year prior to breast cancer diagnosis.

#### Gene-set enrichment pathway analysis

Using gene set enrichment analysis (GSEA) [38], we further examined for functional enrichment of biological pathways associated with radon exposure. We chose gene set databases from the Molecular Signature Database (MSigDB) (http://www.broadinstitute.org/gsea/msigdb/), which included 217 gene sets from BioCarta, 674 gene sets from Reactome and 50 gene sets from Hallmark. We residualized the entire transcriptome matrix on variables that we adjusted for in the single gene model: age at diagnosis (continuous), year of diagnosis (continuous), menopausal status and HT use (post-menopausal not using / post-menopausal using / premenopausal or unknown), BMI (continuous) and surrogate variables. We applied phenotype based permutation (1000 times), we used the Pearson correlation coefficient as the metric for ranking genes. Specifically, we calculated Pearson correlation between radon and each gene in the transcriptome, ranked genes based on their Pearson correlations with radon, and calculated the enrichment score for the list of genes. The enrichment is the maximum deviation from zero encountered in a random walk, and is a measure of whether genes are randomly distributed throughout an *a prior* defined set of genes [38]. We excluded gene sets that contained either less than 15 or greater than 500 genes. We reported gene sets that are significantly enriched at a FDR threshold of less than 0.25.

## Results

### Participant characteristics

One-hundred and eighty-four participants were exposed to high levels of cumulative radon (≥2 pCi/L), while 690 participants were exposed to low levels of cumulative radon (< 2 pCi/L). Mean age at breast cancer diagnosis was slightly younger for participants in the high radon group (mean ± SD = 57.6 ± 11.5 years) compared to the low radon group (mean ± SD = 59.7 ± 11.3 years) (Table 1). We observed comparable median year of diagnosis for both groups. In the high radon group, 30% of participants were postmenopausal and had not used HT, 32% of women were postmenopausal and used HT, while 38% were either premenopausal or did not report HT use. The proportion of women who used HT was comparable in the low radon group. Average BMI was 26.3 kg/m^2^ (SD = 4.9) in the high exposure group and was 26.1 kg/m^2^ (SD = 5.1) in the low radon group. Census tract-level median household income was higher in the low radon group (mean ± SD = 64,480 ± 25,064) compared to the high radon group (mean ± SD = 59,950 ± 18,200). In the high radon group, more than 50% of women lived in the Midwest region at the time of breast cancer diagnosis, while 16% of participants in the low radon group lived in this region. The distribution of radon concentrations in the U.S. is shown in Supplementary Figure 1 (ArcMap 10.4 (Esri, Redlands, CA)) and stratified by ER status and tissue types in Supplementary Figure 2.

**Table 1:** Participant characteristics of women diagnosed with breast cancer in the Nurse’ Health Studies who contributed to tumor data (*N* = 874)

Cumulative average annual radon concentration		
Variable	High (≥2 pCi/L)	Low (<2 pCi/L)
	***N*** = 184	***N*** = 690
Age at diagnosis (year) [mean (SD)]	57.6 (11.5)	59.7 (11.3)
Year of diagnosis [median (IQR)]	2000 (8)	1999 (8)
Cohort [n (%)]		
NHS	100 (54%)	431 (62%)
NHSII	84 (46%)	259 (38%)
Menopausal status / menopausal hormone therapy^a^ [n (%)]		
Postmenopausal not using	56 (30%)	213 (31%)
Postmenopausal using	59 (32%)	256 (37%)
Premenopausal/unknown	69 (38%)	221 (32%)
BMI^a^ (kg/m^2^) [mean (SD)]	26.3 (4.9)	26.1 (5.1)
Pack-year smoked^b^ [n (%)]		
0 (never smoker)	111 (60%)	357 (52%)
≤ 16 pack-year	18 (10%)	71 (10%)
> 16 pack-year	53 (29%)	258 (37%)
Census track median income ($) ([mean (SD)]	59,950 (18,200)	64,480 (25,064)
Region [n (%)]		
Northeast	52 (28%)	233 (34%)
Midwest	102 (55%)	113 (16%)
West	12 (7%)	150 (22%)
South	18 (10%)	194 (28%)

^a^Obtained one cycle before diagnosis

^b^ Pack-year smoking of 16 is the population median among ever smokers

### Single gene analysis

**Fig. 1:**
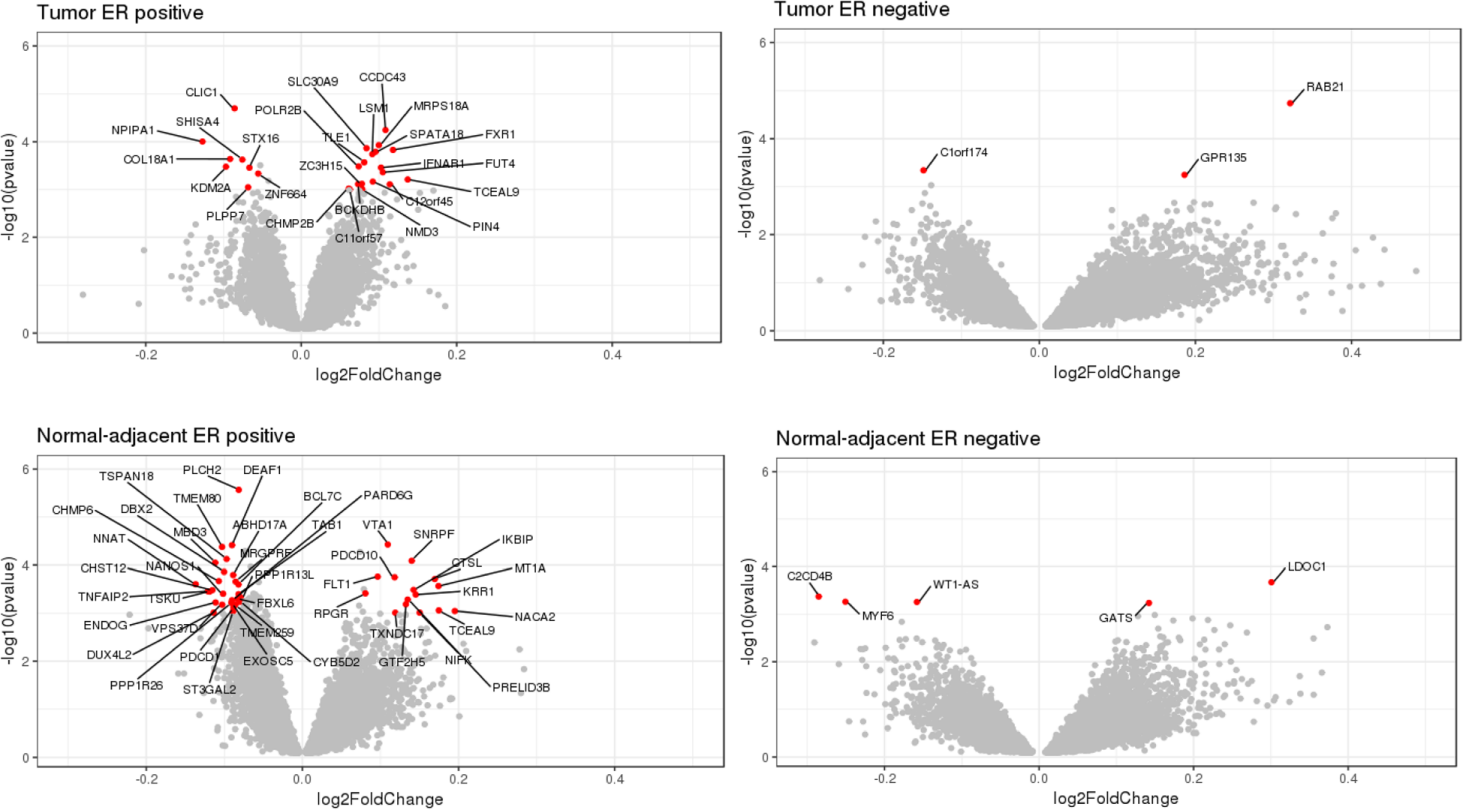
Association between cumulative radon exposures and transcriptome-wide gene expression. Genes in red are those that showed large effect size (i.e., ~ top 5%, which was defined as genes with log fold change larger than the average of the 2.5th and 97.5th percentile) and *p*-value < 0.001 for ER positive tumor (*N* = 707), ER negative tumor (*N* = 167), ER positive normal-adjacent (*N* = 558) and ER negative normal-adjacent (*N* = 129)

In ER positive normal-adjacent samples, cumulative radon exposure was associated with differential expression of *PLCH2* (negative association), a phospholipase gene involved in cellular proliferation (FDR < 5%). We did not find statistically significant associations between cumulative radon exposure and differential gene expression in ER-positive tumor, ER-negative tumor, or ER-negative normal-adjacent samples that met FDR < 5%. In Supplementary Table 1, we presented the top 10 differentially expressed genes sorted by nominal *p* value for tumor and normal-adjacent samples. We observed same signs of directionality, similar magnitudes of effect estimates and similar ranges of *p*-values when we modeled radon as a continuous exposure metric (Supplementary Table 1). Fig. 1 shows differentially expressed genes with relatively large effect size (i.e., ~ top 5%, which was defined as genes with log fold change larger than the average of the 2.5th and 97.5th percentile) and had a nominal *p* value less than 0.001 in tumor and normal-adjacent samples. In ER positive normal-adjacent samples, 7 of the 11 genes that met FDR < 15% also had large effect size (i.e., ~ top 5%).

As sensitivity analyses, we stratified women to never smokers and women smoked > 16 pack-years (population median of ever smokers). Among the top 10 most significant genes identified in the tumor analyses, we observed same signs of directionality and comparable magnitudes of effect estimate in the two stratified populations (Supplementary Table 2). We also observed comparable results when we restricted radon exposure 5 year prior to breast cancer diagnosis (Supplementary Table 1).

### Gene-set enrichment pathway analysis

**Fig. 2:**
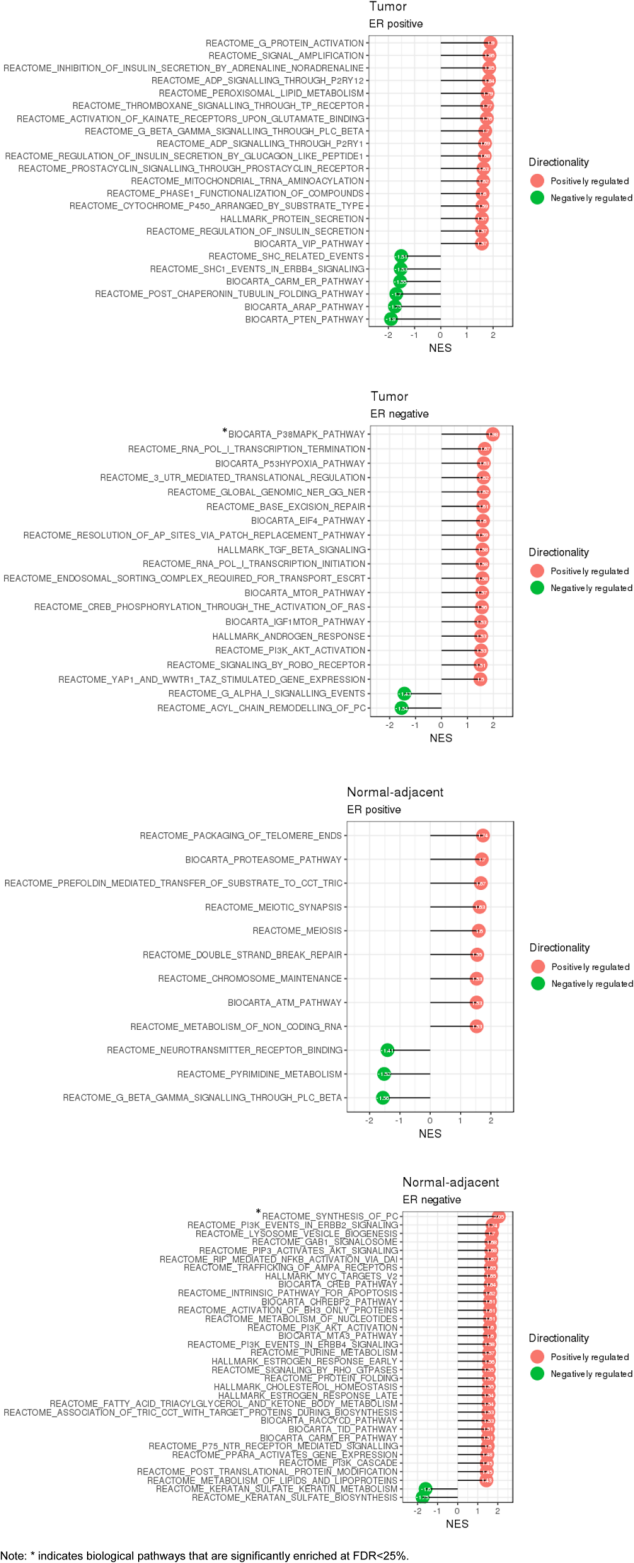
Biological pathways that are significantly enriched at nominal threshold (*p* < 0.05) from gene-set enrichment pathway analysis. NES: normalized enrichment score, which is calculated as the actual enrichment score divide by the mean of enrichment scores against all permutations of the dataset)

**Fig. 3:**
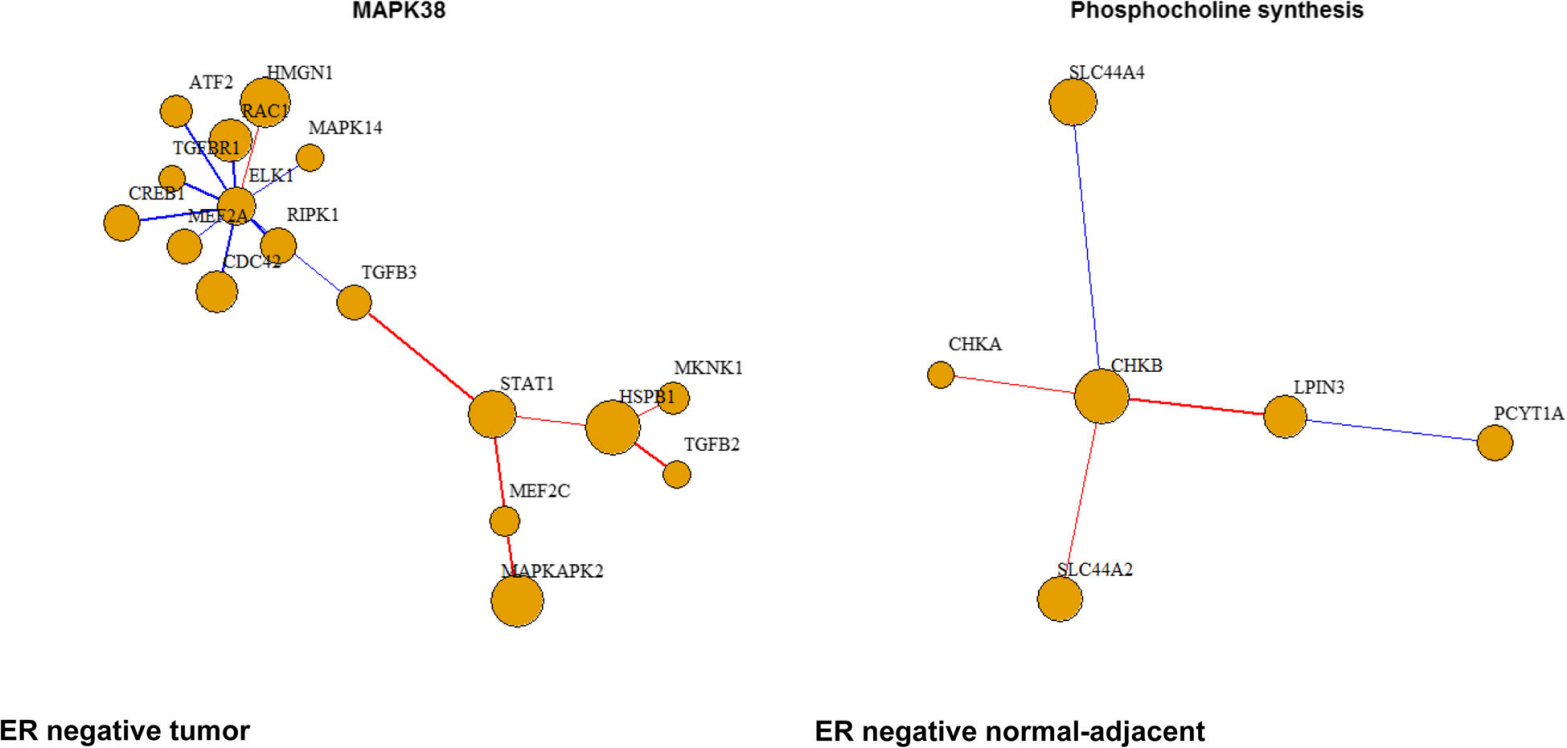
Correlation plots for genes (absolute Pearson correlation > 0.4) that contributed to the enrichment of MAPK38 pathway in ER negative tumor and phosphocholine synthesis in ER negative normal-adjacent samples. Red: positive correlation. Blue: negative correlation. Size of nodes: proportion to the level of expression. Thickness of edges: proportions to the Pearson correlation

At FDR < 25%, we identified two distinct biological pathways significantly enriched in ER negative breast tumor (P38MAPK signaling pathway: up-regulated) and ER negative normal-adjacent samples (synthesis of phosphocholine: up-regulated) with higher cumulative radon exposure (Fig. 2; Supplementary Figure 3). Genes contributing to the core enrichment for P38MAPK signaling included *CREB1*, *TGFBR1*, *MAPKAPK2*, *MKNK1*, *ATF2*, *ELK1*, *MEF2A*, *MAPKAPK5*, *HSPB1*, *RIPK1*, *MEF2C*, *RAC1*, *CDC42*, *MAPK14*, *TGFB3*, *TGFB2*, *STAT1*, and *HMGN1*, while genes contributing to the core enrichment for phosphocholine synthesis included *SLC44A4*, *PCYT1A*, *LPIN3*, *CHKB*, *SLC44A2*, and *CHKA* (Fig. 3). We summarize our key findings for the single gene analysis and pathway analysis in Supplementary Table 3.

## Discussion

To the best of our knowledge, this is the first prospective population-based study investigating the association between low dose radon exposure and gene expression patterns in FFPE breast tumor blocks obtained from invasive breast cancer cases. Overall, we did not see strong evidence of radon exposure being differentially associated with any single gene. However, pathway analyses identified two gene sets (P38MAPK signaling and phosphocholine synthesis) that were significantly enriched after multiple testing adjustment. As we still observed biological effects when radon was modelled at a cutoff (2pCi) below the EPA recommendations (4pCi), this suggests that radon could still possess adverse health consequences at a concentration below the current recommendations.

Radon was among the first human environmental carcinogens identified and was originally described as the chemical associated with “the wasting disease of miners” by Paracelsus in 1567 [39]. To date, it is widely accepted that radon and its progeny contribute significantly to excess lung cancer among underground miners [40, 41]. Although not nearly comparable to the high dose received by uranium miners, several case-control studies conducted in non-occupational settings showed that low dose radon exposure was associated with higher risk of lung cancer [42–46]. In fact, the National Council on Radiation Protection & Measurements (NCRP) estimated approximately 10,000 deaths per year in the U.S., for an average annual radon exposure of 1 pCi/L [40, 47]. Since inhalation is the primary route of exposure, much of the current research for radon focuses on lung cancer as the main health outcome. Nevertheless, based on its lipophilic properties, biokinetic models estimated that deliverable concentrations of radon decay products were detected in the breast and lung at an annual dose of 1000 Bq/L (or 2.7 × 10^4^ pCi/L) [3]. In the NHSII, we previously observed that cumulative radon exposure, even at low doses, was associated with higher risk of ER-negative breast cancer [17]. Consistent with our previous findings, the current work identified mitogenic (P38MAPK signaling: up-regulated) and phosphocholine (synthesis of phosphocholine: down-regulated) pathways that were significantly enriched in ER-negative breast tumor and normal-adjacent tissues (FDR < 25%).

Previous experimental studies have shown that MAPK signaling and phosphocholine biosynthesis played key roles in ionizing radiation induced tumorigenesis. In normal human diploid and tumor cell lines, low dose ionizing radiation could stimulate the MAPK pathway and enhance cell proliferation [48]. Exposure to moderate-to-low dose ionizing radiation also led to the activation of the p42/44 MAPK pathway in animal models [49]. In mammalian cell lines, p38 MAPK has been shown to govern the G2-M transition, and activation of p38 MAPK cascade is required for ionizing radiation induced G2 arrest [50]. Ionizing radiation could also destruct cellular membranes, hydrolyze sphingomyelin to generate phosphocholine, and initiate programmed cell death [51]. In population-based studies, pre-diagnostic phosphocholine has also been shown to be a reproducible biomarker for breast cancer prognosis in multiple cohort studies [52–54].

Single gene analyses identified *PLCH2*, a member of the phospholipase C super family, to be associated with radon exposures in ER-positive normal-adjacent samples. *PLCH2* has essential role for the cleavage of membrane phospholipids, thereby generating second messengers inositol 1,4,5-trisphosphate (PtdIns(4,5)P2) and 1,2-diacylglycerol (DAG) [55, 56]. The 2 s messengers re important for G protein coupled receptor activation. The suppression of *PLCH2* in ER-positive normal adjacent tissue may represents an adaptive/maladaptive response to radon exposures. Stratified analysis by smoking status (never smokers, women who smoked ≤16 pack-years and women who smoked > 16 pack-years) yielded comparable magnitudes of effect estimates, indicating that the association between radon and *PLCH2* did not differ by smoking status. Radon is ionizing radiation and is thought to be associated with both ER-positive and ER-negative breast cancer risks. However, since reproductive and hormonal factors may affect ER-positive breast cancer risk, the association between radon and ER-positive breast cancer risk may be masked. At the molecular level, there may still be molecular alterations associated with both ER-positive and ER-negative tumors. Our study has a number of strengths. To the best of our knowledge, this is the first prospective study to investigate radon exposure with breast cancer biology using transcriptome-wide gene expression data. We included a large number of invasive breast cancer cases. We were able to examine expression in both tumor and normal-adjacent tissues. Previous studies have suggested that the tissue surrounding tumors was morphologically and phenotypically distinct from healthy tissue, and may provide essential information for tumorigenesis [57]. Radon represents a modifiable risk factor with a relatively feasible intervention (radon remediation).

Our study also has a few limitations. Our gene expression data was obtained from FFPE tissue blocks preserved for approximately ~ 20 years (median year of diagnosis was 1999), and RNA degradation maybe of concern. We performed pilot work and showed high correlations between *ESR1*, *PGR*, and *ERBB2* expression with ER, PR, and ER2 immunohistochemistry staining [28, 29]. Our findings are unlikely to be affected by x-ray exposures from mammographic screening, since approximately 80% of women had regular screening (i.e., in each 2 year of the follow up period before diagnosis) [58] and there was little variability by screening intensity/frequency. In our previous study, inverse probability weighting was used to create a hypothetical population in which screening was uncorrelated with all the potential risk factors of breast cancer, and results showed that small changes in effect estimates were observed for breast cancer risk factors [58]. Radon was estimated using a county-level metric that may not actually reflect individual-level exposure and we did not have indoor radon measured individually. Household radon level may differ within a county because of variations in housing characteristics, geology, and radon remediation. Nurses, as health professionals, may be aware of radon as a carcinogen and established remediations. Our radon exposure model has been applied in previous population-based studies of lung cancer, showing expected positive associations [59], which suggests that the model maybe a reasonable proxy for residential radon exposure. Additional research is needed to determine whether our findings can be confirmed in other cohorts, in particular for ER-negative breast cancer cases where sample size was modest. Our single gene and GSEA results need to be confirmed by future studies which include quantitative polymerase chain reactions (qPCR) of core enriched genes and protein biomarkers. Annual radon concentrations, which reflect geological, geographical and meteorological information in the study region, were all below 300 Bq/m^3^. Future studies in elevated radon areas is needed to compare breast cancer characteristics. It would also be important in future work to conduct validation studies to determine the extent to which the radon exposure model is predictive of personal radon exposure.

## Conclusion

To the best of our knowledge, this is the first prospective study to assess the associations between radon exposure and breast tumor and normal-adjacent tissue transcriptomes. Gene set enrichment analysis identified MAPK signaling and phosphocholine biogenesis being upregulated after multiple testing adjustment. Our findings provide further insights into the underlying molecular mechanisms by which low dose radon exposure and may impact breast cancer risk.

## Supplementary information

**Additional file 1.** Table S1. Comparison of signs of directionality, magnitude of effect estimates and p-values when modeling radon as cumulative average (i) up to the year of breast cancer diagnosis as a dichotomized variable (≥2pCi/L vs. < 2pCi/L); (ii) up to the year of breast cancer diagnosis as a continuous variable; and (iii) 5 year prior to breast cancer diagnosis as a dichotomized variable (≥2pCi/L vs. <2pCi/L). Top 10 genes (sorted by p-value) associated with cumulative radon exposures (≥2pCi/L vs. <2pCi/L)—estimated as cumulative averages up to the year of breast cancer diagnosis—by tumor/normal-adjacent and ER status. Table S2. Top 10 genes (sorted by p-value) associated with cumulative radon exposures—estimated as cumulative averages up to the year of breast cancer diagnosis—by tumor/normal-adjacent and ER status. Table S3. Summary of main findings. Sample size: ER positive tumor (N = 707), ER negative tumor (N = 167), ER positive normal-adjacent tissue (N = 558) and ER negative normal-adjacent tissue (N = 129). Figure S1. Map of annual average radon concentration generated from ArcGIS 10.3.1. This figure is not under copyright. Figure S2. Distribution of cumulative radon concentrations for study participants. Figure S3. Gene-set enrichment analysis results (FDR < 25%).

## Data Availability

The data that support the findings of this study are available from the Nurses’ Health Studies, however they are not publicly available. Investigators interested in using the data can request access, and feasibility will be discussed at an investigators meeting. Limits are not placed on scientific questions or methods, and there is no requirement for co-authorship. Additional data sharing information and policy details can be accessed at https://www.nurseshealthstudy.org/researchers.

## Abbreviations

NHS: Nurses’ Health Study
ER: Estrogen receptor
GIS: Geographic information system
EPA: Environmental Protection Agency
SRRS: State Residential Radon Survey
NRRS: National Residential Radon Survey
TMA: Tissue microarray
FFPE: Formalin fixed paraffin-embedded
RMA: Robust multi-array average
ATP: Affymetrix Power Tool
BMI: Body mass index
HT: Hormone therapy
FDR: False discovery rate
GSEA: Gene set enrichment analysis
MSigDB: Molecular Signature Database
NCRP: National Council on Radiation Protection & Measurements
