# Supplementary material for: Low dose environmental radon exposure and breast tumor gene expression
Source: BMC Cancer. 2020 Jul 28;20:695. doi: 10.1186/s12885-020-07184-7 (PMC7385902; doi:10.1186/s12885-020-07184-7)

Supplementary Table 1. Comparison of signs of directionality, magnitude of effect estimates and p-values when modeling radon as cumulative average (i) up to the year of breast cancer diagnosis as a dichotomized variable (≥2pCi/L vs. <2pCi/L); (ii) up to the year of breast cancer diagnosis as a continuous variable; and (iii) 5 year prior to breast cancer diagnosis as a dichotomized variable (≥2pCi/L vs. <2pCi/L). Top 10 genes (sorted by p-value) associated with cumulative radon exposures (≥2pCi/L vs. <2pCi/L)—estimated as cumulative averages up to the year of breast cancer diagnosis—by tumor/normal-adjacent and ER status.

Tumor ER positive (N=707)

		Cum. average of radon up to the year prior to diagnosis dichotomized at 2pCi/L		*Cum. average of radon up to the year prior to diagnosis (continuous)*		*Cum. average of radon 5 year prior to diagnosis dichotomized at 2pCi/L*	
**Gene Name**	**Description**	**logFC**	**p-value**	**logFC**	**p-value**	**logFC**	**p-value**
CLIC1	chloride intracellular channel 1	-0.09	2.00E-05	-0.031	3.26E-04	-0.059	4.41E-04
CCDC43	coiled-coil domain containing 43	0.11	5.67E-05	0.036	1.79E-03	0.079	1.31E-03
NPIPA1	nuclear pore complex interacting protein family member A1	-0.13	9.87E-05	-0.050	4.38E-04	-0.059	1.52E-02
MRPS18A	mitochondrial ribosomal protein S18A	0.10	1.18E-04	0.043	1.40E-04	0.078	1.89E-03
SLC30A9	solute carrier family 30 member 9	0.08	1.36E-04	0.036	1.66E-04	0.064	9.66E-04
FXR1	FMR1 autosomal homolog 1	0.12	1.48E-04	0.040	3.14E-03	0.101	4.77E-04
SPATA18	spermatogenesis associated 18	0.10	1.62E-04	0.033	2.56E-03	0.086	2.12E-04
LSM1	LSM1 homolog, mRNA degradation associated	0.09	1.82E-04	0.034	1.14E-03	-0.043	1.32E-02
COL18A1	collagen type XVIII alpha 1 chain	-0.09	2.29E-04	-0.039	2.98E-04	-0.053	3.91E-03
SHISA4	shisa family member 4	-0.08	2.37E-04	-0.033	2.02E-04	0.062	1.77E-03

Tumor ER negative (N=167)

		Cum. average of radon up to the year prior to diagnosis dichotomized at 2pCi/L		*Cum. average of radon up to the year prior to diagnosis (continuous)*		*Cum. average of radon 5 year prior to diagnosis dichotomized at 2pCi/L*	
**Gene Name**	**Description**	**logFC**	**p-value**	**logFC**	**p-value**	**logFC**	**p-value**
RAB21	RAB21, member RAS oncogene family	0.32	1.82E-05	0.097	4.24E-03	0.287	3.38E-04
C1orf174	chromosome 1 open reading frame 174	-0.15	4.56E-04	-0.045	1.69E-02	-0.177	4.99E-05
GPR135	G protein-coupled receptor 135	0.19	5.68E-04	0.044	6.87E-02	0.187	8.59E-04
ABHD17B	abhydrolase domain containing 17B	-0.14	9.34E-04	-0.043	2.15E-02	-0.123	3.66E-03
SELENOM	selenoprotein M	-0.15	1.36E-03	-0.057	5.19E-03	-0.112	1.87E-02
ZNF789	zinc finger protein 789	0.20	2.12E-03	0.050	8.35E-02	0.154	1.77E-02
CAMK2D	calcium/calmodulin dependent protein kinase II delta	0.27	2.16E-03	0.101	1.10E-02	0.225	1.66E-02
DMWD	dystrophia myotonica, WD repeat containing	0.17	2.18E-03	0.061	1.50E-02	0.196	8.64E-04
CDX2	caudal type homeobox 2	-0.15	2.25E-03	-0.030	1.78E-01	-0.136	7.15E-03
FN3KRP	fructosamine 3 kinase related protein	0.22	2.31E-03	0.064	4.59E-02	0.195	9.98E-03

Normal-adjacent ER positive (N=558)

		Cum. average of radon up to the year prior to diagnosis dichotomized at 2pCi/L		*Cum. average of radon up to the year prior to diagnosis (continuous)*		*Cum. average of radon 5 year prior to diagnosis dichotomized at 2pCi/L*	
**Gene Name**	**Description**	**logFC**	**p-value**	**logFC**	**p-value**	**logFC**	**p-value**
PLCH2	phospholipase C eta 2	-0.082	2.71E-06	-0.026	3.13E-04	-0.083	3.45E-06
VTA1	vesicle trafficking 1	0.109	3.74E-05	0.040	2.86E-04	0.117	1.89E-05
DEAF1	DEAF1, transcription factor	-0.090	3.85E-05	-0.026	4.90E-03	-0.100	7.16E-06

TMEM80	transmembrane protein 80	-0.103	4.20E-05	-0.035	7.04E-04	-0.094	2.70E-04
MAPK8IP3	mitogen-activated protein kinase 8 interacting protein 3	-0.073	5.09E-05	-0.016	3.78E-02	-0.083	7.01E-06
TUBGCP3	tubulin gamma complex associated protein 3	0.074	5.33E-05	0.014	6.51E-02	0.071	1.65E-04
ACAP3	ArfGAP with coiled-coil, ankyrin repeat and PH domains 3	-0.074	6.25E-05	-0.015	5.62E-02	-0.078	3.81E-05
TSPAN18	tetraspanin 18	-0.097	7.50E-05	-0.028	5.40E-03	-0.091	2.79E-04
SNRPF	small nuclear ribonucleoprotein polypeptide F	0.140	8.18E-05	0.041	5.48E-03	0.137	1.71E-04
MBD3	methyl-CpG binding domain protein 3	-0.112	8.95E-05	-0.026	3.04E-02	-0.122	2.65E-05
**Normal-adjacent ER negative (N=129)**							
		Cum. average of radon up to the year prior to diagnosis dichotomized at 2pCi/L		***Cum. average of radon up to the year prior to diagnosis (continuous)***		***Cum. average of radon 5 year prior to diagnosis dichotomized at 2pCi/L***	
**Gene Name**	**Description**	**logFC**	**p-value**	**logFC**	**p-value**	**logFC**	**p-value**
LDOC1	LDOC1, regulator of NFKB signaling	0.30	2.11E-04	0.057	3.35E-03	0.270	7.29E-04
C2CD4B	C2 calcium dependent domain containing 4B	-0.29	4.23E-04	-0.069	4.83E-02	-0.302	8.81E-05
MYF6	myogenic factor 6	-0.25	5.45E-04	0.038	3.39E-02	-0.187	3.53E-03
WT1-AS	WT1 antisense RNA	-0.16	5.51E-04	0.084	3.82E-03	-0.129	7.91E-04
GATS	GATS, stromal antigen 3 opposite strand	0.14	5.77E-04	0.060	8.73E-02	0.153	1.47E-04
ARSG	arylsulfatase G	0.15	1.03E-03	0.080	2.29E-03	0.135	2.79E-03
TBL1X	transducin beta like 1X-linked	0.13	1.09E-03	-0.063	4.60E-02	0.099	7.09E-03
MIF	macrophage migration inhibitory factor (glycosylation-inhibiting factor)	0.20	1.22E-03	0.030	2.09E-01	0.170	1.62E-03
KRTAP10-2	keratin associated protein 10-2	0.22	1.32E-03	0.021	2.20E-01	0.214	7.76E-04
PREX1	phosphatidylinositol-3,4,5-trisphosphate dependent Rac exchange factor 1	0.17	1.35E-03	-0.028	1.62E-01	0.141	8.77E-03

In each regression model, we adjusted for the following covariates selected *a priori*: age at diagnosis (continuous), year of diagnosis (continuous), hormone therapy use (postmenopausal not using / postmenopausal using / premenopausal or unknown), BMI (continuous), Census tract area-level socioeconomic status (continuous), study region (Northeast / Midwest / West / South) and surrogate variables generated from the transcriptome data.

Supplementary Table 2. Top 10 genes (sorted by p-value) associated with cumulative radon exposures—estimated as cumulative averages up to the year of breast cancer diagnosis—by tumor/normal-adjacent and ER status.

**Tumor ER positive**		**Smoking > 16 pack-years N=251**			**Never smoker N=378**		
**Gene Name**	**Description**	**logFC**	**t statistics**	**p-value**	**logFC**	**t statistics**	**p-value**
CLIC1	chloride intracellular channel 1	-0.07	-2.17	3.06E-02	-0.08	-2.70	7.33E-03
CCDC43	coiled-coil domain containing 43	0.13	2.53	1.19E-02	0.10	2.69	7.41E-03
NPIPA1	nuclear pore complex interacting protein family member A1	-0.12	-1.61	1.08E-01	-0.14	-2.99	3.00E-03
MRPS18A	mitochondrial ribosomal protein S18A	0.10	1.62	1.06E-01	0.08	2.50	1.29E-02
SLC30A9	solute carrier family 30 member 9	0.07	1.18	2.39E-01	0.09	3.22	1.41E-03
FXR1	FMR1 autosomal homolog 1	0.08	0.82	4.13E-01	0.12	2.76	6.12E-03
SPATA18	spermatogenesis associated 18	0.05	1.28	2.03E-01	0.12	3.39	7.79E-04
LSM1	LSM1 homolog, mRNA degradation associated	0.06	1.23	2.18E-01	0.12	3.68	2.63E-04
COL18A1	collagen type XVIII alpha 1 chain	-0.03	-0.67	5.01E-01	-0.09	-2.95	3.40E-03
SHISA4	shisa family member 4	-0.08	-2.27	2.42E-02	-0.08	-2.73	6.56E-03

**Tumor ER negative**		**Smoking > 16 pack-years N=60**			**Never smoker N=90**		
**Gene Name**	**Description**	**logFC**	**t statistics**	**p-value**	**logFC**	**t statistics**	**p-value**
RAB21	RAB21, member RAS oncogene family	0.18	1.15	2.56E-01	0.28	3.83	2.47E-04
C1orf174	chromosome 1 open reading frame 174	-0.33	-3.60	6.73E-04	-0.06	-1.18	2.40E-01
GPR135	G protein-coupled receptor 135	0.10	0.93	3.57E-01	0.20	2.92	4.55E-03
ABHD17B	abhydrolase domain containing 17B	-0.18	-1.93	5.93E-02	-0.07	-1.42	1.58E-01
SELENOM	selenoprotein M	-0.32	-3.05	3.52E-03	-0.07	-1.39	1.67E-01
ZNF789	zinc finger protein 789	0.29	2.14	3.68E-02	0.19	2.70	8.38E-03
CAMK2D	calcium/calmodulin dependent protein kinase II delta	0.10	0.55	5.87E-01	0.31	3.12	2.46E-03
DMWD	dystrophia myotonica, WD repeat containing	0.16	1.39	1.71E-01	0.17	2.38	1.96E-02
CDX2	caudal type homeobox 2	-0.20	-1.65	1.05E-01	-0.08	-1.53	1.29E-01
FN3KRP	fructosamine 3 kinase related protein	0.19	1.32	1.91E-01	0.17	1.96	5.28E-02

In each regression model, we adjusted for the following covariates selected *a priori*: age at diagnosis (continuous), year of diagnosis (continuous), hormone therapy use (postmenopausal not using / postmenopausal using / premenopausal or unknown), BMI (continuous), Census tract area-level socioeconomic status (continuous), study region (Northeast / Midwest / West / South) and surrogate variables generated from the transcriptome data.

Supplementary Table 3. Summary of main findings. Sample size: ER positive tumor (N=707), ER negative tumor (N=167), ER positive normal-adjacent tissue (N=558) and ER negative normal-adjacent tissue (N=129).

		ER positive	ER negative
**Tumor**	*Single gene analysis*	None at FDR<5%	None at FDR<5%
	Pathway analysis	None at FDR<25%	1 at FDR<25%
**Normal-adjacent**	*Single gene analysis*	1 at FDR<5%	None at FDR<5%
	Pathway analysis	None at FDR<25%	1 at FDR<25%

Supplementary Figure 1. Map of annual average radon concentration generated from ArcGIS 10.3.1. This figure is not under copyright.

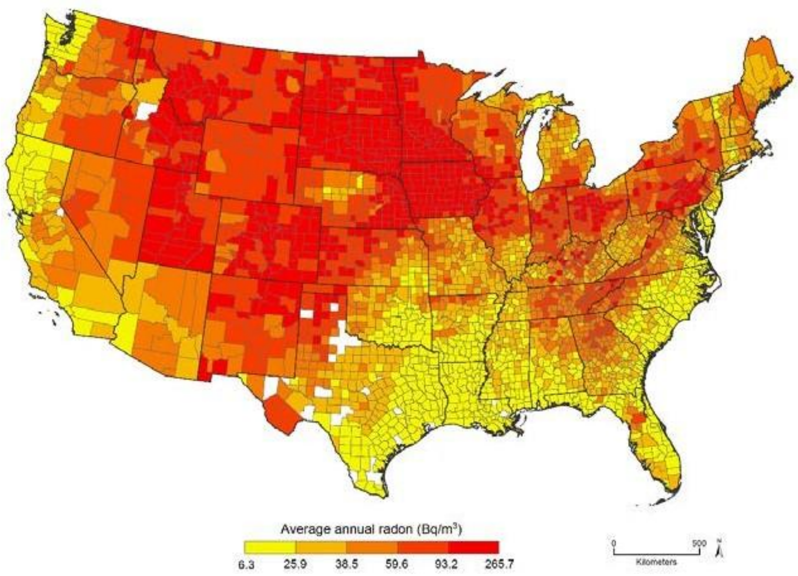

Supplementary Figure 2. Distribution of cumulative radon concentrations for study participants. Sample size: ER positive tumor (N=707), ER negative tumor (N=167), ER positive normal-adjacent tissue (N=558) and ER negative normal-adjacent tissue (N=129).

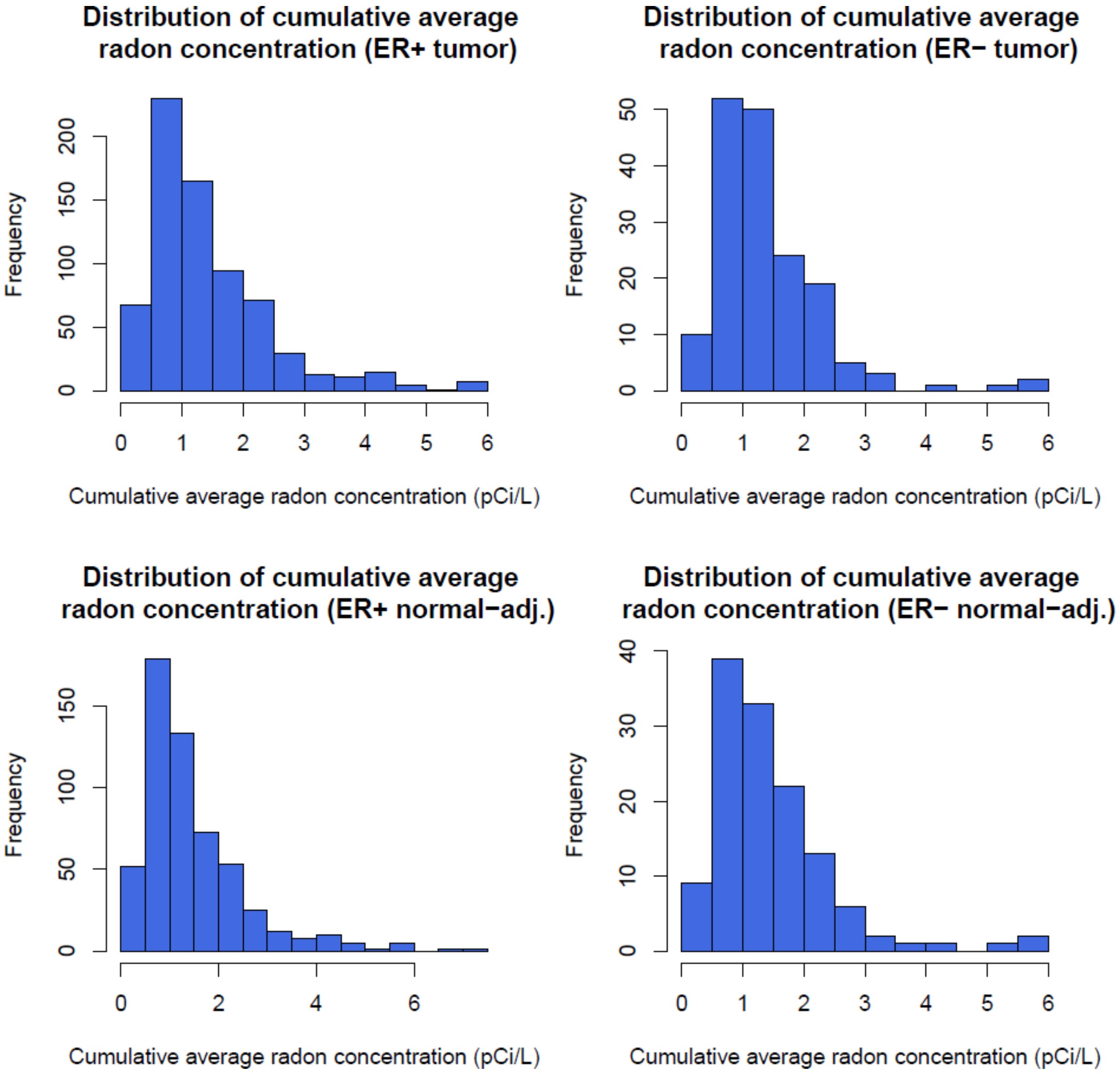

Supplementary Figure 3. Gene-set enrichment analysis results (FDR<25%). In ER negative tumor samples, we identified p38MAPK pathway significantly enriched at FDR<25%. In ER negative normal-adjacent samples, we identified biosynthesis of phosphocholine pathway significantly enriched at FDR<25%.

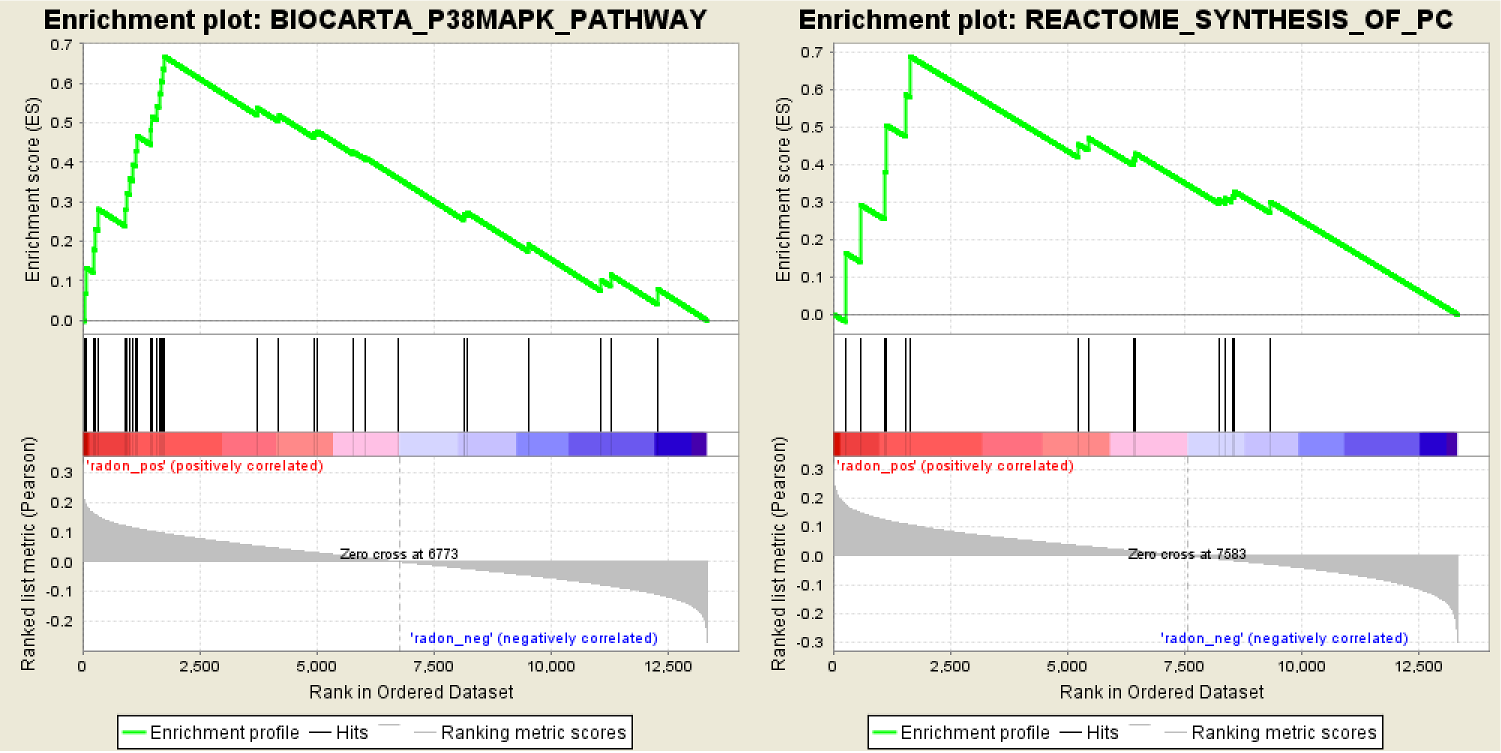

Supplement: Supplementary file 1 — Additional file 1. Table S1. Comparison of signs of directionality, magnitude of effect estimates and p-values when modeling radon as cumulative average (i) up to the year of breast cancer diagnosis as a dichotomized variable (≥2pCi/L vs. < 2pCi/L); (ii) up to the year of breast cancer diagnosis as a continuous variable; and (iii) 5 year prior to breast cancer diagnosis as a dichotomized variable (≥2pCi/L vs. <2pCi/L). Top 10 genes (sorted by p-value) associated with cumulative radon exposures (≥2pCi/L vs. <2pCi/L)—estimated as cumulative averages up to the year of breast cancer diagnosis—by tumor/normal-adjacent and ER status. Table S2. Top 10 genes (sorted by p-value) associated with cumulative radon exposures—estimated as cumulative averages up to the year of breast cancer diagnosis—by tumor/normal-adjacent and ER status. Table S3. Summary of main findings. Sample size: ER positive tumor (N = 707), ER negative tumor (N = 167), ER positive normal-adjacent tissue (N = 558) and ER negative normal-adjacent tissue (N = 129). Figure S1. Map of annual average radon concentration generated from ArcGIS 10.3.1. This figure is not under copyright. Figure S2. Distribution of cumulative radon concentrations for study participants. Figure S3. Gene-set enrichment analysis results (FDR < 25%). [file 12885_2020_7184_MOESM1_ESM.pdf]
